# Raldh1 promotes adiposity during adolescence independently of retinal signaling

**DOI:** 10.1371/journal.pone.0187669

**Published:** 2017-11-02

**Authors:** Di Yang, Charles R. Krois, Priscilla Huang, Jinshan Wang, Jin Min, Hong Sik Yoo, Yinghua Deng, Joseph L. Napoli

**Affiliations:** Graduate Program in Metabolic Biology, Nutritional Sciences and Toxicology, University of California, Berkeley, California, United States of America; Laboratoire de Biologie du Développement de Villefranche-sur-Mer, FRANCE

## Abstract

All-*trans*-retinoic acid (RA) inhibits adipogenesis in established preadipocyte cell lines. Dosing pharmacological amounts of RA reduces weight gain in mice fed a high-fat diet, i.e. counteracts diet-induced obesity (DIO). The aldehyde dehydrogenase Raldh1 (*Aldh1a1*) functions as one of three enzymes that converts the retinol metabolite retinal into RA, and one of many proteins that contribute to RA homeostasis. Female *Raldh1*-ablated mice resist DIO. This phenotype contrasts with ablations of other enzymes and binding-proteins that maintain RA homeostasis, which gain adiposity. The phenotype observed prompted the conclusion that loss of Raldh1 causes an increase in adipose tissue retinal, and therefore, retinal functions independently of RA to prevent DIO. A second deduction proposed that low nM concentrations of RA stimulate adipogenesis, in contrast to higher concentrations. Using peer-reviewed LC/MS/MS assays developed and validated for quantifying tissue RA and retinal, we show that endogenous retinal and RA concentrations in adipose tissues from *Raldh1*-null mice do not correlate with the phenotype. Moreover, male *Raldh1*-null mice resist weight gain regardless of dietary fat content. Resistance to weight gain occurs during adolescence in both sexes. We show that RA concentrations as low as 1 nM, i.e. in the sub-physiological range, impair adipogenesis of embryonic fibroblasts from wild-type mice. Embryonic fibroblasts from *Raldh1*-null mice resist differentiating into adipocytes, but retain ability to generate RA. These fibroblasts remain sensitive to an RA receptor pan-agonist, and are not affected by an RA receptor pan-antagonist. Thus, the data do not support the hypothesis that retinal itself represses weight gain and adipogenesis independently of RA. Instead, the data indicate that Raldh1 functions as a retinal and atRA-independent promoter of adiposity during adolescence, and enhances adiposity through pre-adipocyte cell autonomous actions.

## Introduction

Diverse physiological processes during vertebrate conception, embryogenesis and postnatal development rely on all-*trans*-retinoic acid (RA) signaling [1,2]. Two reactions convert retinol into RA, an autocoid. Retinol dehydrogenases and retinal reductases, of the short-chain dehydrogenase/reductase gene family, catalyze conversion of retinol into retinal, and retinal into retinol [3,4]. At least three isoforms of retinol dehydrogenases (mouse Rdh1/human Rdh16, Rdh10, Dhrs9) and retinal reductases (Dhrs3, Dhrs4, Rdh11) contribute to RA homeostasis under physiological conditions. Retinal dehydrogenases (Raldh1, 2, 3) of the Aldh1 gene family convert retinal into RA. Cells often co-express multiple retinoid metabolizing isoforms; in addition, gene ablations reveal different phenotypes for each, consistent with non-redundant functions [5–13]. Yet, knocking out one isoform can result in compensation by other isoforms, consistent with redundant functions in some locations.

Insulin, RA and intermediary metabolism are interconnected. Insulin regulates RA concentrations by excluding FoxO1 from the nucleus, thereby decreasing RA biosynthesis [14,15]. RA regulates intermediary metabolism by inhibiting adipogenesis, promoting fatty acid oxidation and gluconeogenesis, and inducing UCP1 expression, thereby reducing adiposity [16–18]. Mice fed a high-fat diet (HFD) dosed chronically with RA resist weight gain [19–21]. RA supplementation to HFD-fed mice restricts average weight to ~29 g relative to controls of ~42 g over 8 weeks [22]. Conversely, chronically feeding a vitamin A-deficient HFD increases adiposity [23]. Extending these in vivo observations, ablating the retinol dehydrogenase *Rdh1* facilitates a 33% increase in fat mass in mice fed a low-fat diet, relative to controls [10]. In vitro, RA inhibits preadipocyte differentiation into mature adipocytes in ST13, NIH3T3-LI, 3T3-F44A and C3H10T1/2 cell lines [24–28] through activating RAR [29–31]. Thus, the preponderance of the literature has revealed that RA stimulates energy use and suppresses adiposity.

Raldh1 (encoded by *Aldh1a1*) accesses cellular retinol binding-protein (Crbp1)-complexed retinal to generate RA, is inhibited by apo-Crbp1—an intracellular indicator of retinoid status—and its inhibition decreases liver RA [32–34]. Unexpectedly, therefore, ablation of *Raldh1* affords resistance to diet-induced obesity (DIO) in female mice by impairing adipogenesis [35]. To explain this phenotype, an increase in tissue retinal was postulated to function independently of RA, supporting a conclusion that retinal itself functions as an autocoid. An accompanying hypothesis suggested that low nM RA concentrations induce adipogenesis [36]. Raldh1 has multiple functions, however. It serves as an androgen binding-protein [37–39], and functions in the cornea and lens of mammalian eyes as eta-crystallin [40]. Raldh1 recognizes multiple substrates, including oxazaphosphoranes [41] and aldehyde lipid peroxidation products, such as malondialdehyde and nonenal [42]. Raldh1 also contributes to γ-aminobutyric acid biosynthesis in dopaminergic neurons [43]. Unlike other Raldh, Raldh1 localizes to both cytosol, where conversion of retinal into RA occurs, and in the nucleus, not known to biosynthesize RA [5]. Hence, Raldh1 has multiple functions independent of retinoid metabolism.

We studied mice lacking Raldh1 (KO), and embryonic fibroblasts (MEF) derived from wild-type and KO, to determine the impact of Raldh1 on retinoid concentrations, metabolism and function. The cumulative data indicate that retinoid concentrations in tissues and signaling in preadipocytes do not underlie the KO phenotype. We conclude that Raldh1 has cell-autonomous functions in pre-adipocytes unrelated to retinal metabolism.

## Materials and methods

### Mice

Mouse (*mus musculus*) studies were approved by the UC-Berkeley Animal Use and Care Committee and were done according to AAALAC guidelines. *Raldh1*^+/-^ mice were purchased from Jackson Laboratory (Stock #012247). Upon arrival, mice were fed a vitamin A-sufficient purified diet with 4 IU vitamin A/g (AIN-93G diet) and 7% fat, as recommended for rodents by the National Research Council [44]. This diet will be designated as an LFD, even though it contains a recommended amount of fat for rodents. Heterozygotes were interbred, yielding homozygous null mice (KO) that were interbred and maintained on the LFD. C57Bl/6J mice were used as controls. Mice were fed the LFD at least 10 generations to normalize the vitamin A tissue content [45]. Where noted, mice were fed the LFD modified to contain 50% fat-derived calories (HFD). Mice were euthanized with isoflurane confirmed by cervical dislocation. All efforts were made to minimize discomfort.

### Quantification of retinoids

Tissue and cellular RA values were quantified by LC/MS/MS [46], with modified LC conditions. LC was done using a Suplex pkb-100 column (Supelco, 2.1 x 250 mm, 5 μm particles). LC was eluted with a gradient of 80% acetonitrile/20% water/0.1% formic acid for 3 min, followed by a linear gradient to 95% acetonitrile/5% water/0.1% formic acid over 9 min, held for 4 min, returned by linear gradient to the original mobile phase over 1 min and held 8 min, all at 0.4 mL/min. Tissue and primary cell retinal values were measured after O-ethyloxime derivatization, using an ultra-high performance liquid chromatography MS/MS assay with a 5 fmol lower limit of detection [47]. Retinol was measured with a LC/UV assay [48].

### MEF isolation and differentiation

Embryos (e13.5–14) were separated from maternal tissue and yolk sacs, heads and internal organs were removed, and bodies were minced, digested with 2 ml 0.25% trypsin/1 mM EDTA for 15 min at 37°C, re-suspended in 8 ml of UV irradiated growth medium, consisting of Dulbecco's modified Eagle's medium (DMEM; Life Technologies #11995073), 10% bovine calf serum (BCS; ATCC 30–2030), and 100 U/ml penicillin/streptomycin (Gibco BRL). Cells were centrifuged 5 min at 1,000 × *g* and cultured in 6-well plates (8 x 10^3^ cells/cm^2^) at 37°C. Upon reaching confluence (designated as dd0), the medium was replaced with differentiation-induction medium consisting of UV-irradiated growth medium containing 0.5 mM methylisobutylxanthine, 1 μM dexamethasone, 0.85 μM insulin, 100 nM rosiglitazone and 10% (vol/vol) bovine calf serum. Cells were exposed to differentiation-induction medium for 3 days, then cultured in UV-irradiated growth medium with 0.85 μM insulin and 100 nM rosiglitizone until harvest. The medium was renewed every other day. Each well contained cells from a single embryo. MEF experiments were repeated with cells from different dams.

RNA was harvested from cells before (dd0) and after differentiation (dd4 to dd7). Total RNA was extracted by TRI Reagent (Sigma Aldrich, #T9424). One μg total RNA was used for reverse transcription (iScript, #1708891). qPCR was performed with a Bio-RAD CFX Connect Real-Time Detection System. qPCR Primers. Primers used were: *Adipoq* (Mm00456425_m1), *Aldh1a1* (Mm00657317_m1), *Aldh1a2* (Mm00501306_m1), *Aldh1a3* (Mm00474049_m1), *Bmp2* (Mm01340178_m1), *Bmp4* (Mm00432087_m1), *Cebpa* (Mm.PT.58.30061639.g), *Cyp26B1* (Mm00558507_m1), *Dhrs3* (Mm00488080_m1), *Dhrs9* (Mm00615706_m1), *Ebf1* (Mm.PT.58.30999400), *Fabp4* (Mm00445878_m1), *Gusb* (Mm01197698_m1), *Klf2* (Mm00500486_g1), Lipe (Mm00495359_m1), *Pnpla2* (Mm00503040_m1), *Pparg* (Mm00440940_m1), *Rarb* (Mm01319677_m1), *Rdh10* (Mm00467150_m1), *Tbp* (Mm01277042_m1), *Zfp423* (Mm00677660_m1), Zfp521 (Mm00521009_m1).

Oil red O staining was done with cells (dd7) washed twice with PBS and fixed 1 hr with 10% neutral buffered formalin in PBS. Cells were washed three times with water and stained with oil red O (6 parts of 0.6% oil red O in isopropanol and 4 parts water) for 30 min. Excess stain was removed by washing with water 5 times. Stained cells were dried. Spectrophotometric quantification was done by dissolving stained oil droplets in isopropanol for 10 min and reading absorbance at 510 nm.

### Statistics

Data are presented as mean ± S.E. and were analyzed by unpaired, two-tailed student’s t-tests or by one-way or two-way ANOVA, as appropriate. Experiments were repeated 2 to 3 times. Representative experiments are shown.

## Results

### Male and female KO both resist weight gain

**Fig 1:**
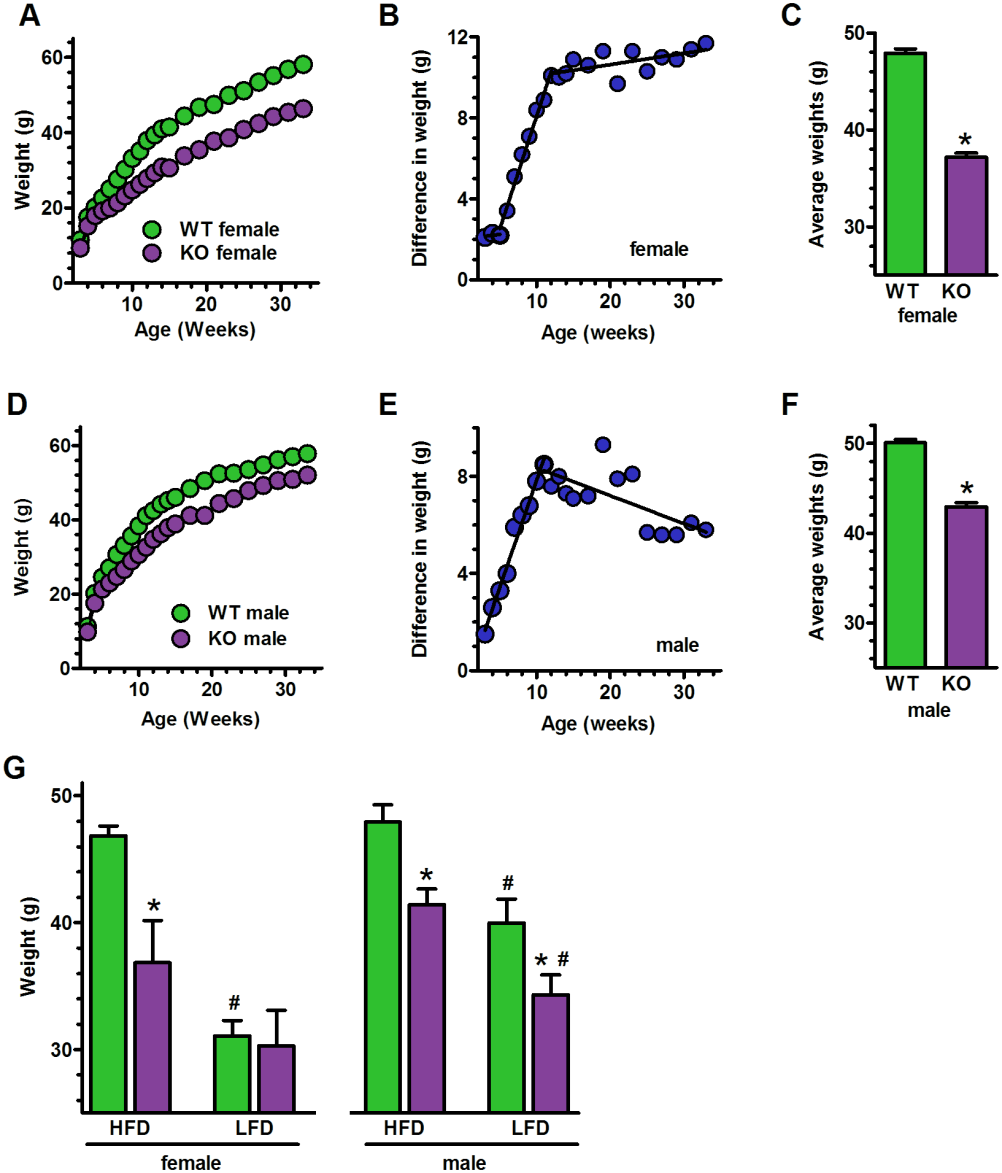
Male and female mice respond differently to a LFD vs a HFD. WT green, KO purple. (A) Weights of female mice fed a HFD beginning at weaning (n = 23 WT, 25 KO). Two-way ANOVA: genotype, p<0.001; age, p<0.001; Bonferroni posttests p<0.001, 7 through 33 wks. (B) Differences in weights between female WT and KO with age. (C) Average weights of all female WT and KO mice from 12 to 33 –wks-old: *p<0.0001 (n = 286 WT, 324 KO). (D) Weights of male mice fed a HFD beginning at weaning (n = 16 WT, 20 KO). Two-way ANOVA: genotype, p<0.001; age, p<0.001; Bonferroni posttests p<0.05 at 6 wks. Weeks 7 through 33 p<0.001. (E) Differences in weights between male WT and KO with age. (F) Average weights of all male WT and KO mice from 11 to 33-wks-old: *p<0.0001 (n = 221 WT, 223 KO). (G) Weights of 26-wk-old mice transferred from a LFD to a HFD beginning at 13-wks-old or continued with a LFD until 26-wks-old: *p<0.05 compared to WT (n = 7–10 per group); #p<0.005 compared to HFD.

Male and female KO pups fed a HFD resisted weight gain starting shortly after weaning and continuing until 10 weeks old, i.e. during adolescence (Fig 1A and 1D) [49].

After adolescence, weight differences did not diverge further (Fig 1B and 1E). Therefore, weights from 10 to 33-week-old mice were averaged to enable calculating steady-state differences between WT and KO of 10.7 ± 0.6 g for females and 7.1 ± 0.6 g for males (Fig 1C and 1F).

In a different experiment, mice fed a LFD for 13 weeks were divided into two groups. One was fed the LFD for a further 13 weeks and the second was switched to a HFD for a further 13 weeks (Fig 1G). Because the amount of vitamin A was the same in each diet, this tested the impact of a LFD vs a HFD on weight gain in WT vs KO mice. Male KO resisted weight gain even when fed a LFD for the entire 26 weeks, weighing ~6 g less than WT; females fed the LFD did not differ from WT. Thus, *Raldh1*-ablation restricts weight gain in males regardless of dietary fat, but restricts weight gain in females only when fed a HFD. Male and female KO transferred to the HFD weighed ~6 and 10 g less than WT, respectively, showing that Raldh1 maintains protection when a HFD is introduced later in life.

### Tissue retinoids do not underlie resistance to weight gain

**Fig 2:**
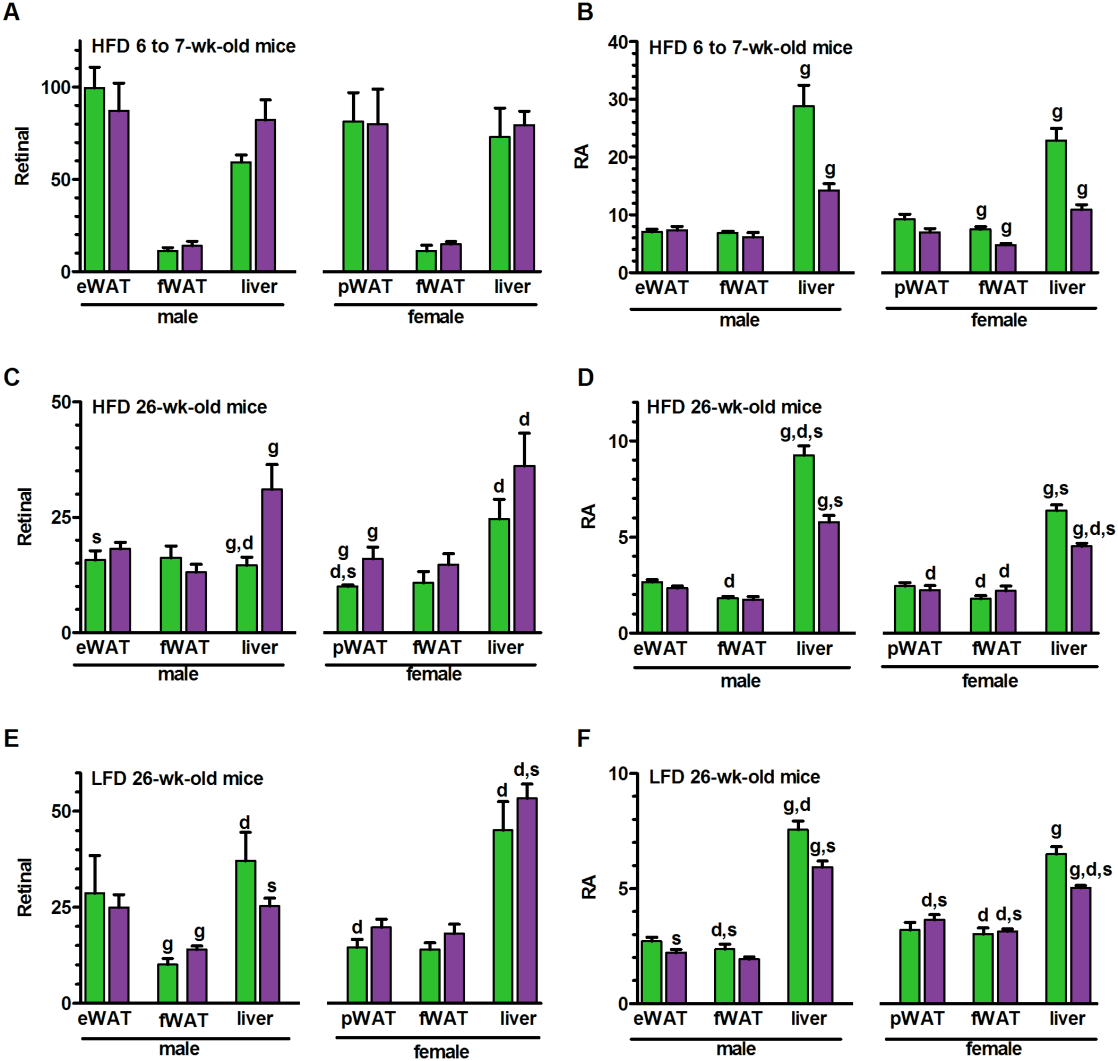
Tissue retinoid concentrations. Mice were fasted overnight then refed 4–4.5 hr: units, pmol/g tissue; green bars, WT; purple bars, KO. (A) Retinal in 6 to 7-wk-old mice fed a HFD for 2 weeks (8–9 mice/group). (B) RA in the same mice as A. (C) Retinal in 26-wk-old mice fed a LFD since weaning and switched to a HFD beginning at 13-wk-old (7–9 mice/group). (D) RA in the same mice as C. (E) Retinal in 26-wk-old mice fed a LFD since weaning (6–11 mice/group). (F) RA in the same mice as E. eWAT, epididymal WAT; fWAT, femoral WAT; pWAT, parametrial WAT. ^**g**^Significant difference between genotypes in the same tissue, sex and diet. ^**d**^Significant difference between diets (LFD vs. HFD, C vs E and D vs F) in the same tissue, sex and genotype. ^**s**^Significant difference between sexes in the same tissue, genotype and diet. ^g, d, s^p<0.05.

If either retinal or RA were primary drivers of resistance to weight gain in the *Raldh1*-null mouse, tissue retinoid concentrations should differ prior to emergence of weight differences, and sex differences should correlate with the different responses to dietary fat. At the onset of weight differences at 7-wks-old, prompted by the HFD, we did not detect differences in retinal between genotypes of either sex in liver or in any adipose depot (Fig 2A). These data do not support the previously proposed mechanism that *Raldh1*-ablation increases adipose retinal to levels that would activate nuclear receptors. In this group of mice, atRA in KO did not change relative to WT in male epididymal WAT (eWAT) or femoral WAT (fWAT), or in female parametrial WAT (pWAT). atRA did decrease, however, in male and female liver of KO and in female fWAT of KO, relative to WT (Fig 2B). A decrease in adipose tissue RA should have increased adipogenesis, followed by weight gain [10,22]. Therefore, RA does not cause resistance to weight gain in the *Raldh1*-null mouse.

We next examined the effects of an HFD on retinal and RA tissue concentrations in 26-wk-old mice fed a LFD until 13-wk-old and then switched to a HFD. The retinal concentration was ~2-fold greater in livers of these older KO males fed a HFD compared to WT, but was unchanged in eWAT or fWAT (Fig 2C). Retinal increased ~30% in older KO female pWAT, but did not change significantly in fWAT or liver. In the same group of mice, liver RA was 2–3 pmol/g tissue lower in KO of both sexes, but did not change in eWAT or f/pWAT (Fig 2D). These data do not support retinal or RA as causal for the phenotype in adipose, because: 1) retinal and RA concentration changes occurred after emergence of the phenotype; 2) retinal concentrations did not correlate with weight gain in either sex or with differences in weight gain between sexes; 3) a decrease in liver RA is not consistent with resistance to DIO. The 6 pmol/g increase of retinal in female KO pWAT is insufficient to force occupation of RAR, because retinoids lacking an acidic group at C15 have RAR μM binding affinities, i.e. at least 3 orders of magnitude lower than those with a carboxylate function [50–53].

Next, we compared retinal and RA concentrations in both genotypes of 26-wk-old mice fed a LFD from weaning. Retinal increased in male fWAT by 4 pmol/g, but no changes in retinal were observed in any of the female tissues (Fig 2E). RA did not change in fat pads of either sex, but decreased in livers of both sexes by ~23% or <2 pmol/g (Fig 2F). These changes occurred after the onset of resistance to weight gain in the male, and did not correlate with the phenotype.

By comparing Fig 2C to 2E, an assessment can be made of the HFD effect on retinal. A HFD decreased retinal in WT male liver and in livers of both WT and KO females relative to the LFD. The HFD did not affect retinal concentrations in pWAT or fWAT of KO females. If adipose retinal were a driving force for resistance to DIO by females, its concentrations should have increased in both fat pads of female KO mice fed a HFD. Comparing Fig 2D to 2F shows that, where changes occurred, RA decreased in fWAT of males and in all three tissues of female HFD-fed mice. The impact of the HFD on retinal and RA concentrations does not support involvement of either retinoid as causing resistance to weight gain in mice fed a HFD.

### Cell autonomous differentiation of MEF into adipocytes

**Fig 3:**
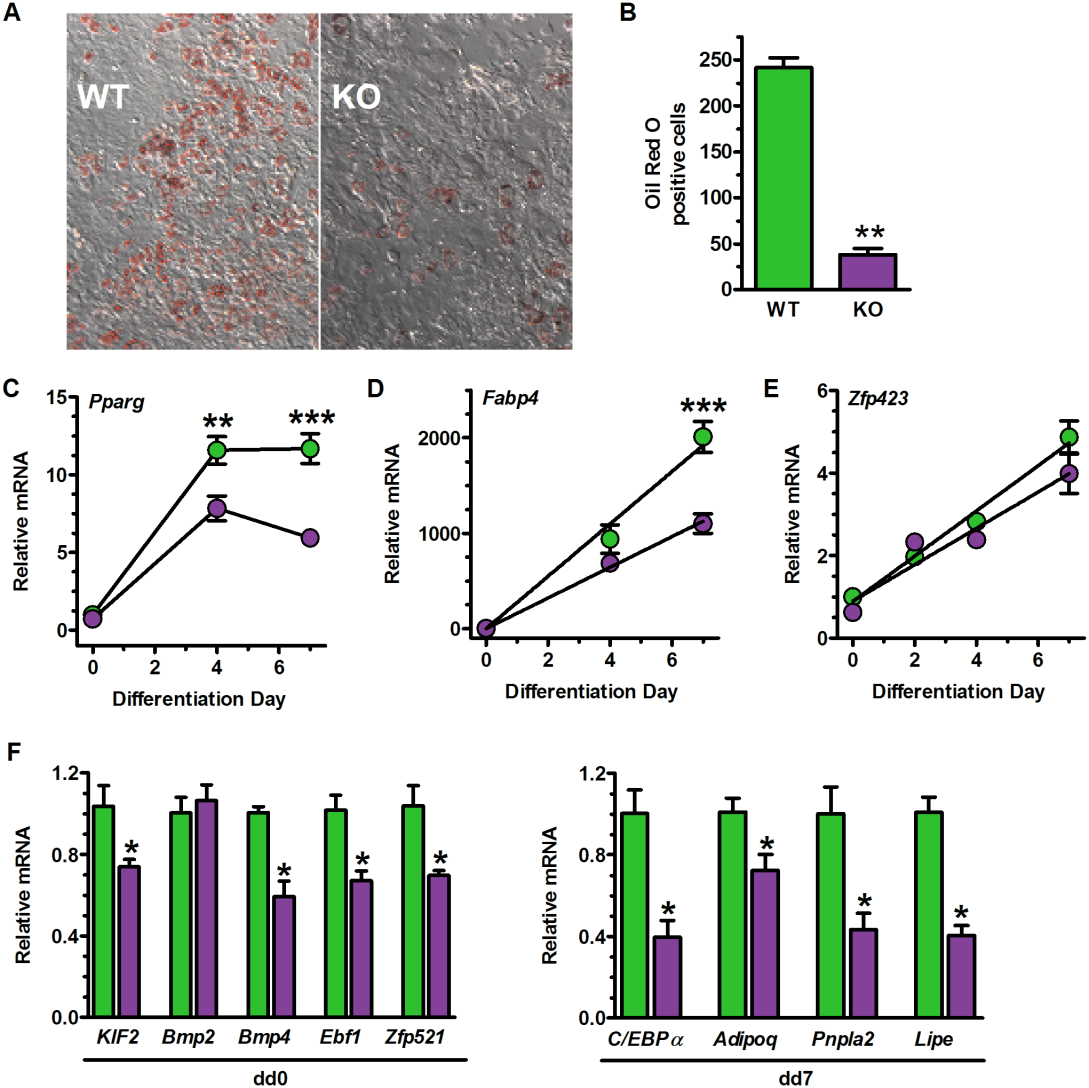
Raldh1 ablation causes cell-autonomous impairment in adipogenesis. WT, green; KO purple. (A) MEF differentiation into adipocytes. Representative images from three independent experiments of cells stained with oil red O at the end of dd7. (B) Quantification of oil red O staining of cells illustrated in A: n = 8 WT embryos, 14 KO embryos: **p<0.01. (C) *Pparg* mRNA expression during adipogenesis. Two-way ANOVA: genotype p<0.0001; dd p<0.001. Bonferroni post-tests of dd (WT vs KO): **p<0.01, ***p<0.001. (D) *Fabp4* (*Ap2*) mRNA expression during adipogenesis. Two-way ANOVA, genotype p<0.0002; dd p<0.0001. Bonferroni post-test of dd (WT vs KO): ***p<0.001. (E) *Zfp423* mRNA expression during adipogenesis. Two-way ANOVA: genotype p = 0.054; dd p<0.0001. (F) Expression of pre and post-adipogenesis gene markers: n = 4, *p<0.04 by student’s t-test.

MEF derived from KO did not differentiate efficiently into adipocytes (Fig 3A). Oil red O staining at the end of differentiation day 7 (dd7) revealed an ~85% decrease in KO lipid accumulation (Fig 3B). Reduction of *Pparg* and *Fabp4* mRNA confirms an underlying deficiency in adipogenesis (Fig 3C and 3D). Expression of *Zfp423* remained similar between WT and KO throughout adipogenesis (Fig 3E). Adipose differentiation in most preadipocyte cell lines, however, does not correlate with *Zfp423* expression [54]. Decreases in expression of transcription factors that regulate adipogenesis (Klf2, Ebf1, Zfp521) or designate mature adipocytes further indicate abnormal differentiation of KO MEF (Fig 3F) [55–57].

### Dose dependent RA arrest of MEF adipogenesis

**Fig 4:**
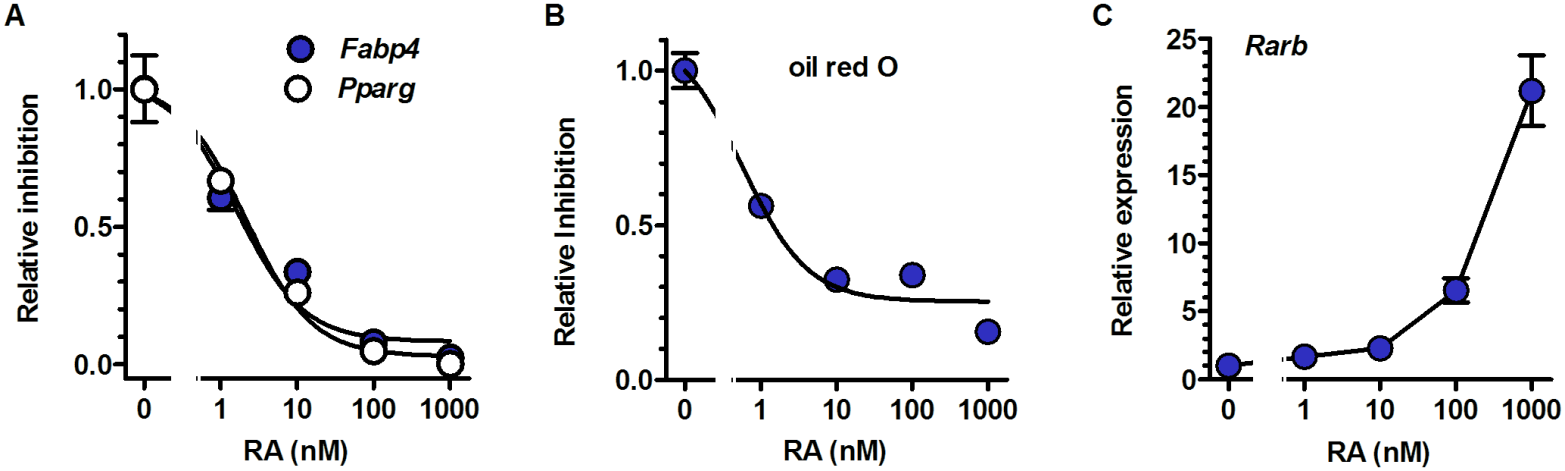
atRA inhibits MEF adipogenesis. (A) *Fabp4* and *Pparg* mRNA expression at the end of dd7 in WT MEF. MEF were treated with increasing doses of RA from dd0 through dd7. The medium was changed every 24 hr with new RA added to maintain continued RA presence. Data from three embryos were normalized to values of 1 for untreated cells. (B) MEF from WT as treated in A were stained with oil red O at the end of dd7. Data from three embryos were averaged. (C) Effects of graded doses of atRA on *Rarb* mRNA in MEF at the end of dd7. Data were fit by non-linear regression analyses.

RA inhibited MEF adipogenesis with IC_50_ values (averages of two experiments) of 3.4 nM for *Pparg* mRNA, 2.1 nM for *Fabp4* mRNA, and 0.6 nM for oil red O staining (Fig 4A and 4B). One nM RA caused a 33–40% decrease in *Pparg* and *Fabp4* mRNA. Increasing RA doses increased mRNA of the RA target *Rarb*, as expected (Fig 4C) [58–60]. Our results from primary pre-adipocytes are consistent with data generated using the established pre-adipocyte cell line NIH3T3-L1, in which RA inhibits differentiation with IC_50_ values between 1 and <10 nM [61,62]. These results do not support the conclusion that RA concentrations lower than 10 nM induce adipogenesis [63], which was used to propose a mechanism for the KO phenotype [36].

### MEF differentiation affects mRNA levels of retinoid metabolic enzymes

**Fig 5:**
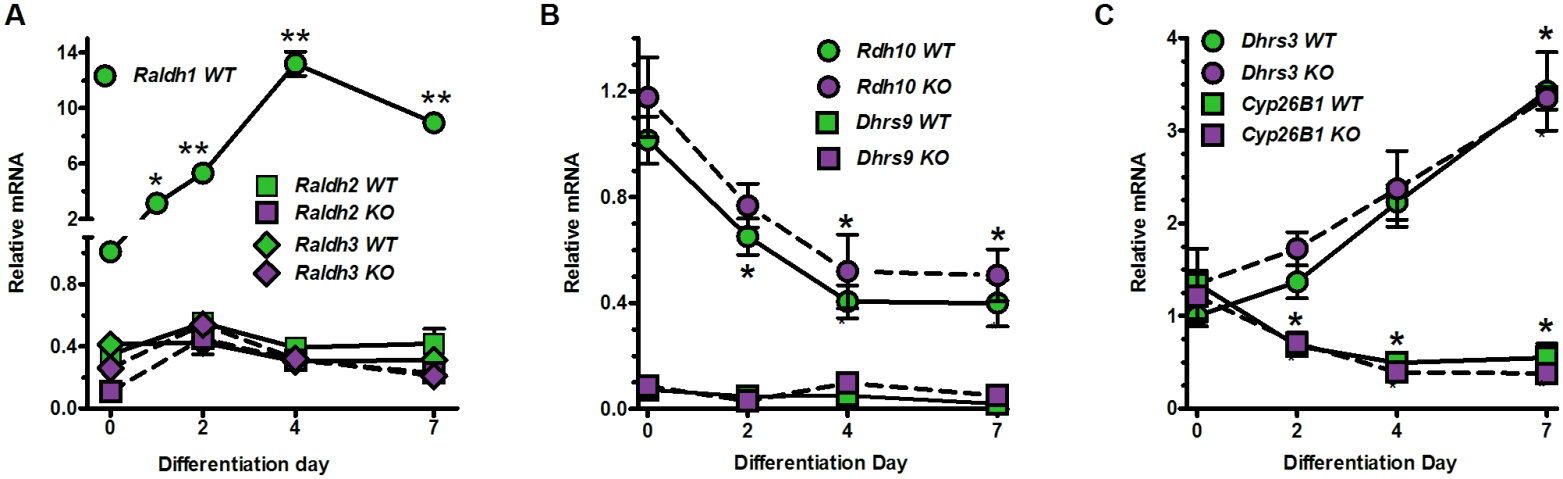
Changes in retinoid metabolism genes during MEF differentiation. (A) Raldh mRNA expression during MEF differentiation into white adipocytes, normalized to Raldh1 on dd0. One-way ANOVA: differentiation effect on Raldh1 mRNA, p<0.0001. Dunnett’s multiple comparison: *p<0.05; **p<0.001 vs dd0. (B) *Rdh* mRNA expression in WT and KO MEF were normalized to *Rdh10* on dd0. One-way ANOVA: differentiation effect on *Rdh10* mRNA, WT and KO, p<0.02. Dunnett’s multiple comparison: *p<0.02 vs dd0. (C) *Dhrs3* and *Cyp26B1* mRNA expression in WT and KO MEF. *Dhrs3* and *Cyp26B1* mRNA were normalized to *Dhrs3* on dd0. One-way ANOVA: differentiation effect on *Dhrs3* and *Cyp26b1* for both WT and KO, p<0.001. Dunnett’s multiple comparison: p<0.02 vs dd0. (A-C) n = 3–4 embryos/data point.

WT MEF express Raldh1 mRNA ~3-fold more intensely than the mRNA of Raldh2 and Raldh3 on dd0 (Fig 5A). Raldh1 mRNA increases up to ~13-fold during differentiation, peaking on dd4, and remains high. Raldh2 and Raldh3 mRNA do not increase as cells differentiate. In KO MEF, neither Raldh2 nor Raldh3 mRNA compensate for the absence of Raldh1, consistent with no need to replace Raldh1 to maintain RA biosynthesis. MEF express Rdh10 mRNA most intensely on dd0 with greater than 60% decreases by dd4 in WT and KO, consistent with decreasing RA to allow pre-adipocyte differentiation (Fig 5B). mRNA of the retinol dehydrogenase Dhrs9 remains low throughout differentiation, whereas the mRNA of the retinal reductase Dhrs3 increases ~3-fold (Fig 5C). MEF express Cyp26b1 mRNA on dd0 with decreases during differentiation, but do not express Cyp26a1 or Cyp26c1. These data show that the RA metabolons in primary MEF, and in adipocytes derived from MEF, do not compensate for the absence of Raldh1.

### Raldh1 absence does not decrease MEF conversion of retinol into RA

**Fig 6:**
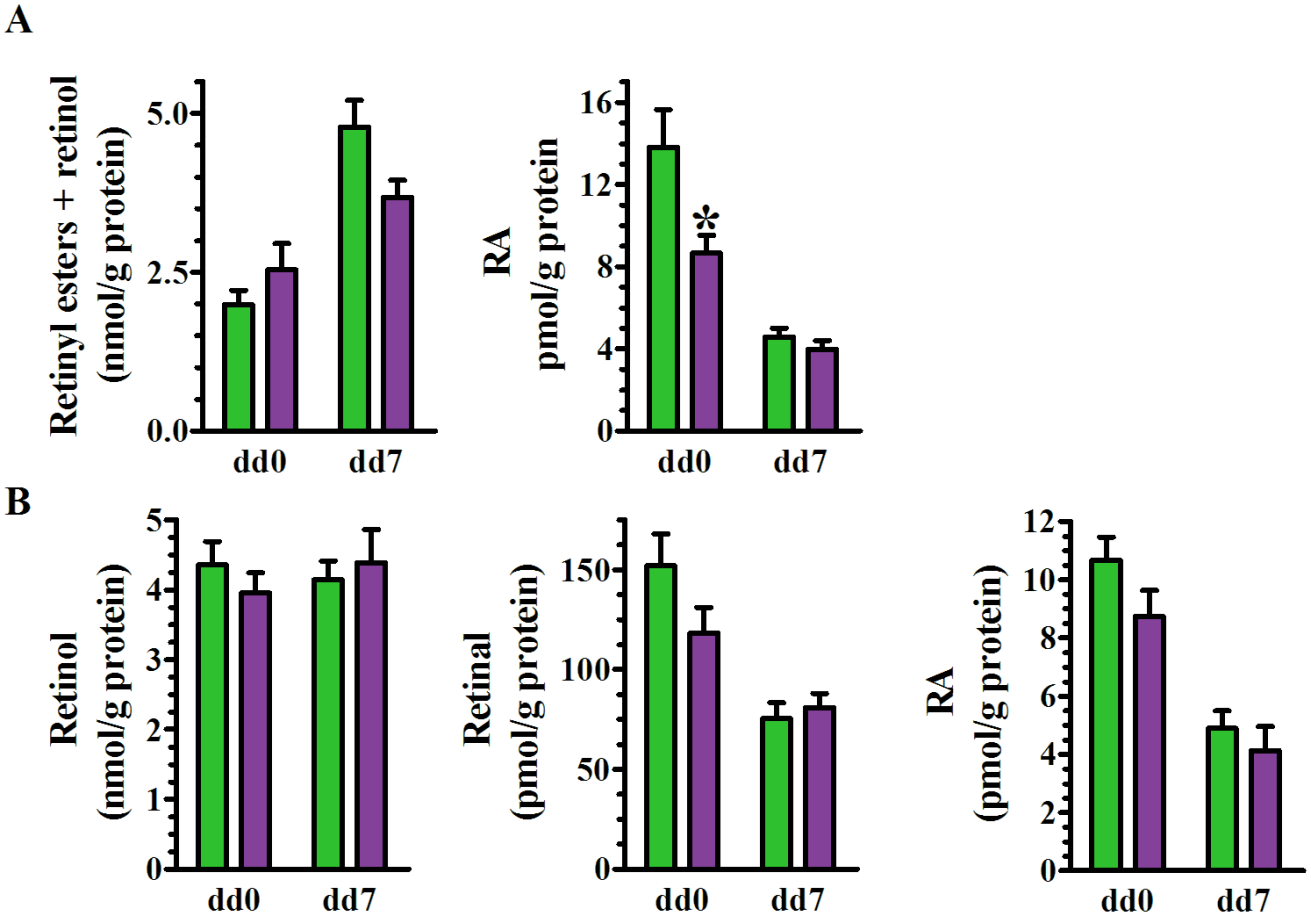
Retinoid metabolism during MEF differentiation into adipocytes. WT green, KO purple. (A) Retinal conversion into RE plus retinol vs RA before (dd0) and after (dd7) MEF differentiation into adipocytes. Cells were incubated 2 hr with 100 nM retinal: SE of 2 experiments, n = 13–14 embryos/genotype. Two-way ANOVA: RE + retinol, genotype p>0.4, dd p<0.0001; RA, genotype p = 0.01, dd p<0.0001. Bonferroni posttest: *p<0.01 relative to WT; (B) Retinol conversion into retinal and RA. Cells were incubated 2 hr with 250 nM retinol, SE of 3 experiments, n = 13–14 embryos/genotype. Left panel, retinol recovered. Two-way ANOVA: retinal, genotype p>0.2, dd p<0.0001; RA, genotype p~0.1, dd p<0.0001. Bonferroni posttest: no significant differences WT vs KO.

The rate of retinal reduction—the sum of retinyl esters (RE) and retinol—outpaced dehydrogenation >330-fold on dd0 and >1600-fold on dd7 and did not differ with genotype (Fig 6A). RA biosynthesis from exogenous retinal in KO decreased ~37% on dd0 vs WT, but not on dd7. Differentiation itself decreased retinal conversion into RA by 50 to 70% and eliminated differences between WT and KO. These data reflect changes in *Rdh10* and *Dhrs3* mRNA, but not in *Raldh1* mRNA. The data also indicate that Raldh1 contributes to RA biosynthesis in MEF on dd0 from exogenous retinal, but is not the only Raldh to do so, and does not contribute to RA biosynthesis from exogenous retinal on dd7.

Retinol serves as the usual substrate for RA biosynthesis in vivo. Cell-associated retinol uptake did not differ in MEF by genotype or differentiation day (Fig 6B). Retinol supported retinal and RA synthesis in MEF to the same extent regardless of genotype. Differentiation (dd7) caused a ~42% decrease in retinal synthesis on dd7 and a ~54% decrease in RA. Thus, ablating *Raldh1* does not increase concentrations of retinal generated from retinol in MEF, and RA synthesis from retinol reflects a decrease in Rdh10 and an increase in Dhrs3 mRNA, but not the increase in Raldh1 mRNA.

### A RAR pan-antagonist does not prevent the phenotype caused by the absence of Raldh1

**Fig 7:**
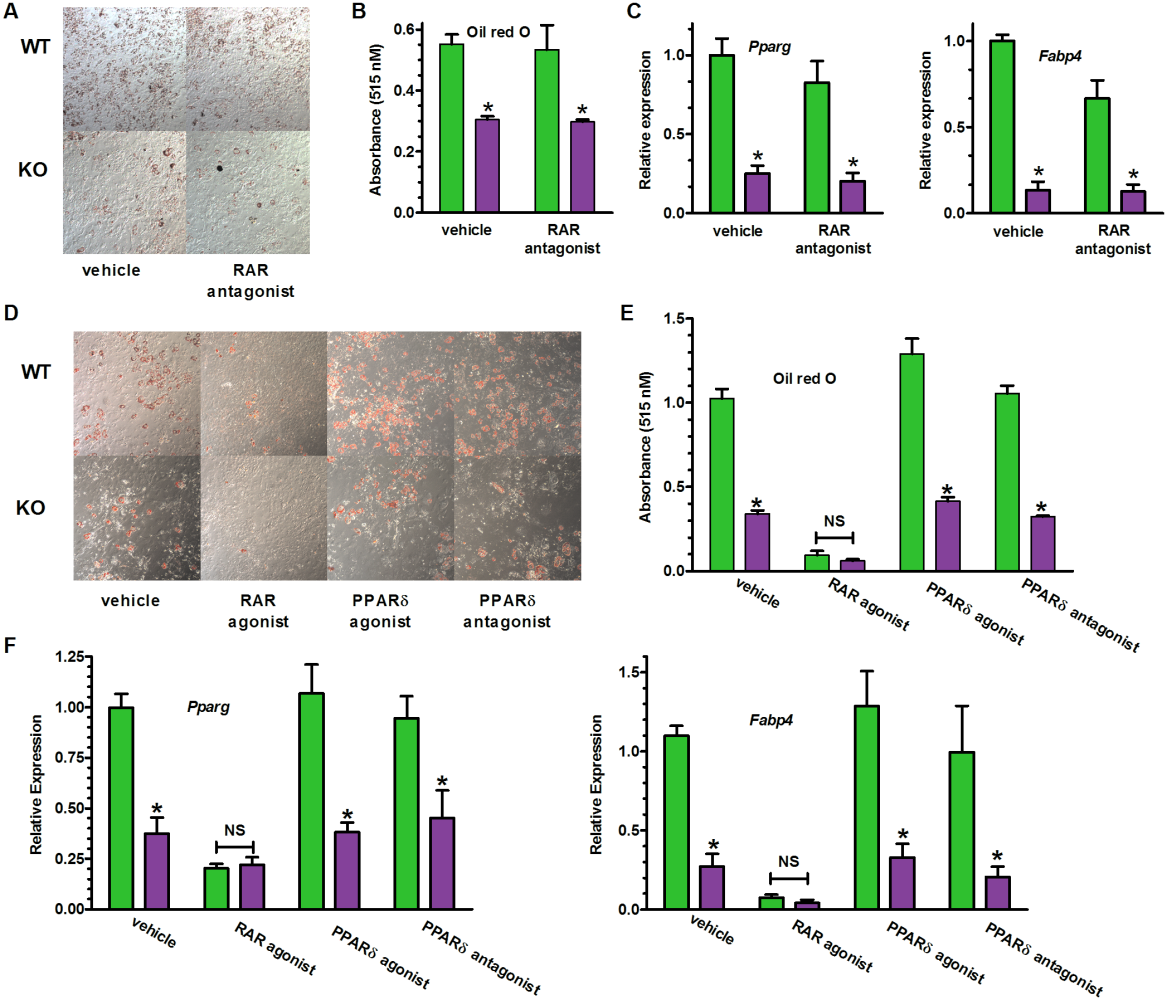
Neither activation nor antagonism of RAR and PPARδ reverses the KO phenotype. (A) MEF were treated with DMSO (vehicle) or an RAR pan-antagonist (200 nM AGN193109) from dd0 through dd7, and were stained with oil red O at the end of dd7. B, C, E, F: WT green, KO purple. (B) Quantification of RAR pan-antagonist treatment in A: two-way ANOVA, genotype *p<0.0006. *Bonferroni posttests p<0.05 WT vs KO. (C) Adipocyte genes in MEF treated with the RAR pan-antagonist in A. Two-way ANOVA, genotype *p<0.0001. *Bonferroni posttests p<0.01, WT vs KO. (D) Effects of an RAR pan-agonist (100 nM TTNPB), PPARδ agonist (100 nM GW0742), or PPARδ antagonist (1 μM GSK3787). Cells were treated from dd0 through dd7, and were oil red O stained at the end of dd7. (E) Quantification of D. Two-way ANOVA, genotype differences between WT and KO for all treatments, p<0.0004; impact of RAR agonist, p<0.0001. Bonferroni posttests: vehicle WT vs KO, *p<0.001. (F) Expression of adipocyte genes in MEF treated as in D. Two-way ANOVA, genotype differences between WT and KO, p≤0.002; impact of RAR agonist, p<0.001. Bonferroni posttests: vehicle WT vs KO, *p<0.05 WT vs KO, except for RAR agonist. (A-F) n = 3 embryos/group.

The RAR pan-antagonist AGN193109 did not rescue impaired differentiation of KO MEF, as it would have if the phenotype were driven by activation of RAR. Nor did the RAR pan-antagonist prevent differentiation of WT (Fig 7A and 7B). Ability of KO MEF to sequester lipids was ~60% lower than WT, regardless of treatment with vehicle or the RAR pan-antagonist. Decreased *Pparg* and *Fabp4* expression confirm arrested differentiation of KO and lack of reversal by RAR antagonism (Fig 7C). The RAR pan-agonist TTNPB suppressed differentiation in MEF to the same level regardless of genotype, indicating that both WT and KO respond to RAR with equivalent sensitivity (Fig 7D, 7E and 7F). RA also activates PPARδ [64], but neither an agonist (GW0742) nor an antagonist (GSK3787) of PPARδ affected differentiation in either genotype.

## Discussion

This work shows that absence of Raldh1 enables male mice to resist weight gain regardless of dietary fat content. The data presented here provide new insight into the sex-specific effects of Raldh1, revealing a modest difference between males and females fed a HFD, but a profound difference when feeding a LFD, i.e. the amount of dietary fat normally fed to mice.

Resistance to weight gain by the *Raldh1*-null mouse manifests mostly during adolescence in both sexes fed a HFD. This too was unexpected. These data suggest complex interactions among Raldh1 and multiple hormones and metabolites, because heightened responses to stressors via hormones and the hypothalamic-pituitary-adrenal axis occur during adolescence. The *Raldh1*-null mouse should be useful for studying mechanisms of weight-gain during adolescence.

The totality of data reveals that removing Raldh1 does not ameliorate weight gain by altering retinal or RA tissue concentrations. No differences in liver or adipose retinal concentrations occurred at the onset of weight gain promoted by a HFD. Changes in RA involved only decreases, which should have enhanced adipogenesis. The restricted and mostly modest increases in retinal occurred after weight differences emerged. Retinal concentrations did not correlate with weight gain, considered in context of genotype, diet or sex. Nor were concentrations of retinal (pmol/g tissue) at any time sufficient to activate RAR, because retinal has a micromolar *k*_d_ for nuclear receptors. Conversely, decreases in RA, if having an effect, should have exacerbated weight gain. It is important to note that we quantified retinoids with robust, peer-reviewed assays based on LC/MS/MS. These assays were validated for use with adipose and liver, and had low fmol lower limits of quantification.

Loss of Raldh1 did not decrease RA biosynthesis by MEF from retinol, nor increase the retinal concentration, suggesting a modest Raldh1 contribution to retinal homeostasis and the RA concentration in MEF. This outcome is consistent with lack of compensation by altered expression of other retinoid metabolon enzymes, and by inverse correlation of Raldh1 mRNA with RA biosynthesis during MEF differentiation. These data, along with more intense expression of Raldh1 during adipogenesis as RA biosynthesis decreases, suggests that Raldh1 serves a purpose in MEF other than generating RA.

The effect of Raldh1 in primary pre-adipocytes was cell autonomous, with KO MEF remaining responsive to the anti-adipogenic properties of the RAR agonist TTNPB. Neither RAR nor PPARδ antagonism enabled KO MEF to differentiate efficiently. These results do not support retinal, RA, RAR or PPARδ causing resistance to weight gain in KO, nor concluding that retinal functions as an autocoid. This evidence should direct attention from retinoids concerning the mechanism of Raldh1 related to weight gain, and prompt study of its retinoid-independent functions during adolescence. The non-retinoid functions of Raldh1 also should be considered concerning the poor prognosis of breast and prostate cancer, and the use of Raldh1 as a marker of cancer stem cells [65,66].
